# Polidocanol induced tubal occlusion in nonhuman primates: immunohistochemical detection of collagen I-V^☆^^☆☆^

**DOI:** 10.1016/j.contraception.2016.07.003

**Published:** 2016-11

**Authors:** Ov D. Slayden, Dong Ock Lee, Shan Yao, Jeffrey T. Jensen

**Affiliations:** a Division of Reproductive & Developmental Sciences, Oregon National Primate Research Center, Beaverton, OR, 97006, USA; b Center for Uterine Cancer, National Cancer Center, Gyeonggi-do, 410-769, Republic of Korea; c Department of Obstetrics & Gynecology, Oregon Health & Science University, Portland, OR, 97239, USA

**Keywords:** Permanent contraception, Sterilization, Nonhuman primate, Tubal occlusion, Collagen, Fallopian tube

## Abstract

**Objective:**

Intrauterine administration of polidocanol foam (PF) can create fallopian tube occlusion in nonhuman primates. The objective of this study was to determine if PF-induced tubal obstructions contain collagen in the extracellular matrix.

**Study design:**

We compared tissue samples of the intramural fallopian tube obtained from previous studies evaluating the effects of intrauterine infusion of 5% PF 2–12 weeks after treatment. Serial sections of the intramural portion of the fallopian tube obtained from representative treated (rhesus macaques, *n*=7; baboon, *n*=11) and untreated control (macaque, *n*=3; baboon, *n*=5) animals were stained with hematoxylin and eosin to identify tubal occlusion and by immunohistochemistry for collagens Col-I, Col-III and Col-IV. Descriptive results are summarized.

**Results:**

Control animals exhibited histologically normal fallopian tubal epithelium with no staining for Col-1, light staining for Col-III and Col-V in the lamina propria and Col-IV distributed evenly in the extracellular matrix of the lamina propria. Treatment with PF resulted in acute tissue damage confined to the intramural tube; no epithelial damage or occlusion occurred in the tubal isthmus or ampulla. Blockade of the intramural tube demonstrated fibrosis with the epithelium replaced with extracellular matrix that stained strongly for Col-I, Col-III, Col-IV and Col-V. Col-II was undetectable.

**Conclusion:**

Tubal blockage induced by PF resulted in loss of normal epithelium and accumulation of collagens Col-I, Col-III, Col-IV and Col-V at the site of obstruction. The presence of dense collagen staining supports the hypothesis that PF infusion creates lasting tubal obstructions.

**Implications:**

This study demonstrates that PF-induced tubal occlusion results in deposition of collagens suggesting the potential for a more lasting blockade. The structural nature of this occlusion supports the development of intrauterine administration of PF as a nonsurgical method of permanent contraception.

## 1 Introduction

There remains an unmet need for effective contraception worldwide. In developing countries, greater than 24% of married women wanting contraception lack modern methods [1]. A shift away from sterilization, the most effective method, toward reversible methods, appears to have contributed to increases in unintended pregnancies in countries with limited resources [2]. In contrast, permanent contraception by tubal ligation is one of the most common contraceptive methods utilized in the United States. A recent review of data from the 2011–2013 National Survey of Family Growth revealed that, in the United States, 44.2% of women aged 35–44 years choose permanent female contraception compared to 7.6% choosing long-acting reversible methods. Even in younger women aged 25–34 years, 21.7% choose permanent female contraception compared to 16.5% who choose long-acting reversible methods [3].

Surgical sterilization is a costly procedure that requires a trained medical provider, medical equipment and dedicated procedure rooms. Women in many lesser developed countries, in particular, rural areas, cannot easily access the procedure [4]. Development of inexpensive nonsurgical methods of permanent contraception would greatly improve family planning for many women in low-resource settings.

One approach to nonsurgical permanent contraception is the intrauterine administration of chemical agents that cause fallopian tube sclerosis and occlusion. The most studied chemical agent used to create tubal sclerosis was quinacrine hydrochloride [5], [6]. Development of this approach was stopped due to concerns over the potential toxicity of quinacrine [7]. A safer alternative sclerosing agent is polidocanol (hydroxy-polyethoxy-dodecane), a synthetic long-chain fatty alcohol that has a high therapeutic index of safety [8]. Polidocanol solutions (0.5% and 1%) are approved by the Food and Drug Administration in the United States for the treatment of uncomplicated “spider” and “reticular” varicose veins up to 3 mm in diameter. We recently demonstrated that transcervical intrauterine infusion with polidocanol foam (PF) can create tubal occlusion in nonhuman primates [9], [10].

Tubal obstruction induced by PF results in an increase of fibrosis surrounding the intramural tube where it passes through the uterine myometrium [10]. We hypothesized that PF-stimulated tubal occlusion will be long lasting (permanent) if the composition of the fibrosis zone consists of extracellular matrix of collagen bridging the walls of the tube. The goal of this study was to characterize, by immunohistochemistry, collagen deposition in the intramural zone of the nonhuman primate oviduct after PF therapy.

## 2 Methods

### 2.1 Animals

We have previously reported the general care, characteristics and outcomes of animals used in our initial experiments evaluating PF for permanent contraception [9], [10]. For this study, we examined tissue samples obtained from adult cycling female rhesus macaques housed at the Oregon National Primate Research Center (ONPRC) and baboons housed at the Southwest National Primate Research Center (SNPRC) used in these prior experiments. (All studies were reviewed and approved by the ONPRC and SNPRC Institutional Animal Care and Use Committees, in accord with the National Institutes of Health Guidelines for Care and Use of Laboratory Animals.) Necropsies and tissue collection were carried out on by veterinary pathologists at the ONPRC and by the pathology services unit at SNPRC after euthanasia by the attending veterinarian.

### 2.2 Animal treatment

Macaques [10] and baboons [9] received intrauterine administration of 5% PF as previously reported. Briefly, to administer PF, a small silicone balloon catheter (Model J-CHSG-503000, Cook, Bloomington, IN, USA) was placed transcervically into the uterine cavity of the sedated female. A 5% polidocanol solution in sterile physiologic buffered saline was mixed with air to create foam [11] that was infused through the catheter directly into the uterine lumen. Control females included untreated animals in whom the transcervical procedure was not successful (*n*=1 macaque; *n*=5 baboons) and animals that received transcervical infusion of methylcellulose foam (*n*=2 macaques). Our previous studies indicate that methylcellulose alone does not cause permanent tubal blockade (10).

### 2.3 Tissue collection

Necropsy followed at 2, 4 and 7 weeks after PF infusion of macaques (*n*=7) and at 5, 9, 11 and 12 weeks after PF infusion of baboons (*n*=11). During collection, the uterus was bisected, and the cornua with the intermural portion of fallopian tube was dissected as a single block, fixed in 10% neutral-buffered formalin and processed by standard histologic technique. Serial sections (5 μm) were cut with a HM325 Rotary Microtome (Thermo Scientific, Waltham, MA, USA) mounted on poly-l-lysine-coated slides (Fisher Scientific) and 1/5 slides was stained with hematoxylin and eosin (H&E) to identify sites of tubal occlusion. We used samples from both occluded and nonoccluded tube for immunohistochemistry.

### 2.4 Immunohistochemistry

Immunohistochemistry was conducted using a standardized technique [12] at room temperature unless otherwise noted. Slides were deparaffinized in xylene, rehydrated stepwise in ethanol:H_2_O and then rinsed in deionized water. Antigen retrieval was carry out on section by heating in citrate buffer (BioGenex, Fremont, CA, USA) in a pressure cooker at 15 psi for 10 min. The sections were washed with phosphate-buffered saline (PBS; Sigma Chemical Co, St. Louis, MO, USA), incubated in 3% hydrogen peroxide in methanol for 30 min and then treated with species-specific normal serum (ABC-kit, Vector Laboratories Inc., Burlingame, CA, USA) for 20 min, rinsed with PBS and then incubated overnight at 4°C with primary antibodies diluted in 1% bovine serum albumin (Cat. A3803 Sigma) in PBS.

Collagen Col-I was detected with Clone I-8H5 (10 μg/mL; ICN Biomedicals, Aurora, OH, USA), Col-II was detected with Clone 6B3 (2 μg/mL; Abcam, Cambridge, MA, USA), Col-III was detected with Clone III-53 (2 μg/mL; ImmunO™, Takaoka, Toyama, Japan), Col-IV was detected with goat polyclonal antibody NBP1-26549 (1 μg/mL; Novus Biologicals, Littleton, CO, USA) and Col-V with Clone 13F6 (5 μg/mL; LifeSpan BioSciences, Santa Fe Springs, CA, USA). Antibody concentrations were determined by performing a series of serial antibody dilutions. IHC controls included no primary antibody and nonspecific primary antibody.

After overnight incubation with primary antibodies, the slides were rinsed with PBS containing 0.075% nonionic detergent BRIJ (Sigma) and then blocked with the appropriate normal serum for 20 min. Secondary biotinylated antibodies were added and incubated (30 min) at room temperature. After rinsing with PBS containing BRIJ, the sections were incubated in avidin-biotin peroxidase reagent solution (Vector Laboratories Inc., Burlingame, CA, USA) for 60 min and rinsed in PBS with BRIJ followed by Tris buffer (38 mmol/L Tris, pH 7.60; Invitrogen, Carlsbad, CA, USA). Color was developed with 0.025% 3,3′-diaminobenzidine (Dojindo Inc., Richmond, VA, USA) in Tris buffer and 0.03% hydrogen peroxide for 10–15 min according to the status of staining and then rinsed in Tris buffer and deionized water repeatedly. The sections were then placed in 0.026% osmium tetroxide for 1 min and rinsed with deionized water. For counterstaining, Meyer's hematoxylin was used for 1 min. After dehydration with ethanol and clearing with xylene, the slides were mounted with Permount. Digital images were taken using a Zeiss Axiolmager A.I microscope (Carl Zeiss, Inc., Oberkochen, Germany) and a Leica DFC 480 camera (Leica, Wetzler, Germany).

## 3 Results

### 3.1 Tubal blockade

**Fig. 1:**
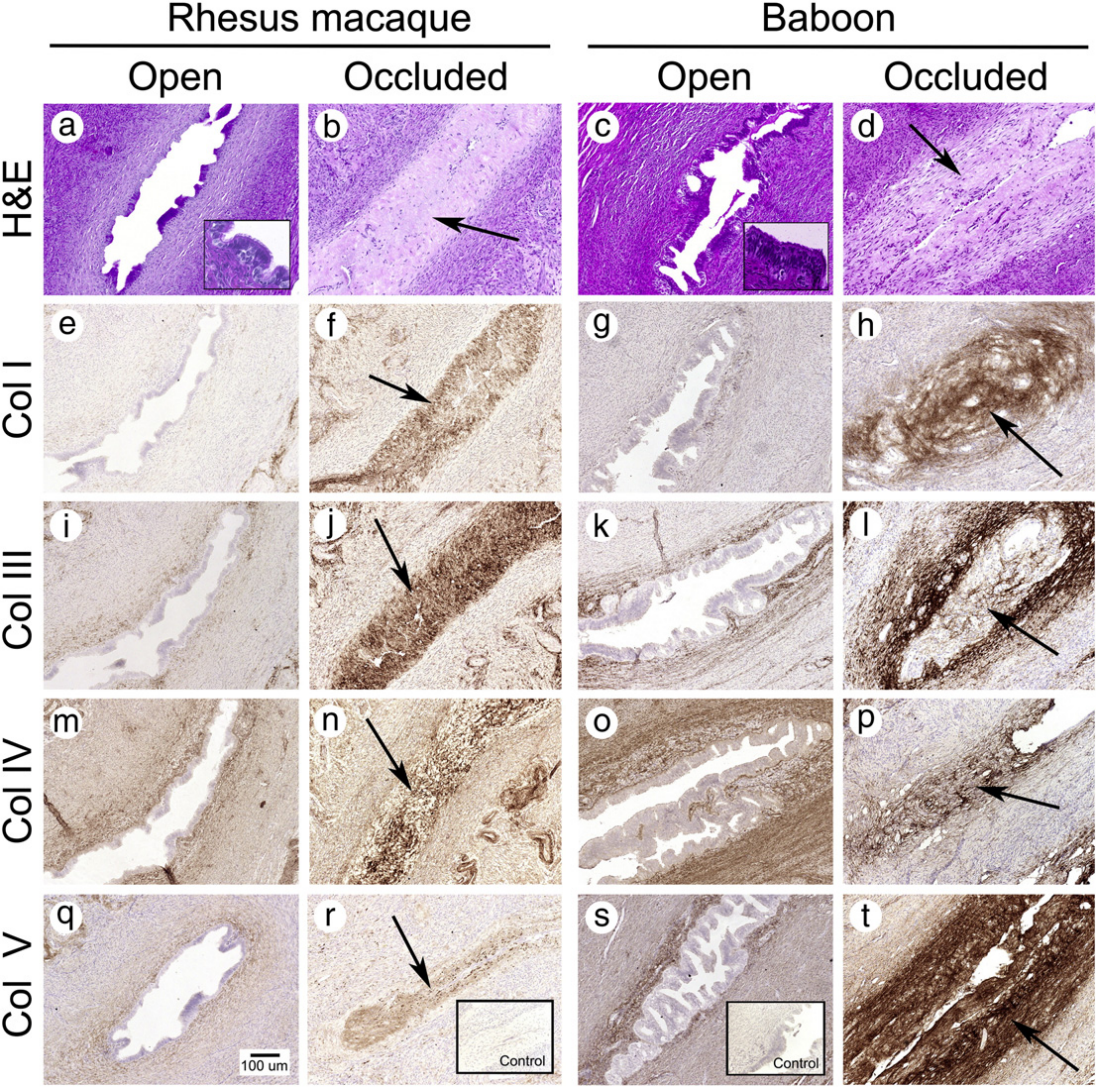
Photographs showing H&E staining and immunostaining for Col-I, Col-III, Col-IV and Col-V in the rhesus macaque and baboon intramural tube. Images of open fallopian tube (a, c, e, g, i, k, m, o, q and s) were obtained from control animals. Occluded tube (b, d, f, h, j, l, n, p, r and t) were from animals treated with 5% PF. Arrows indicate fibrotic response. All images were captured at original magnification ×200. Insets (frame r and s) show negative control with an irrelevant antibody. Treatment with PF increased collagen immunoreactivity in the lamina propria of the intramural tube.

Fig. 1a–d shows photomicrographs of paraffin sections stained with H&E from the intramural tube from rhesus macaques and baboons. Completely blocked portions of the tube were identified histologically as a hyalinizing occlusion of the tubal lumen associated with a loss of tubal epithelium. None of the control animals developed hyalinizing occlusion. In contrast, 4/5 rhesus and 5/7 baboons receiving PF developed complete bilateral blockage. The remaining animals receiving PF failed to develop hyalinizing blockage and were either similar to controls or with partial blockade (not shown). We observed occlusion in the intramural fallopian tube of the PF-treated animals. We observed no occlusions or increase in hyalinization in the isthmic or ampullary portion of the tube.

### 3.2 Collagen staining in the intramural fallopian tube

Fig. 1e–t shows photographs of the intramural tube immunostained for Col-I, Col-III, Col-IV and Col-V. Staining with an irrelevant IgG (negative control) is shown in Fig. 1r and s. In control animals, the epithelium of the intramural tubal epithelium was intact, and there was no collagen staining in the epithelial cells. Control animals also showed no staining for Col-1 in the lamina propria. In these animals, light staining for Col-III and Col-V was observed in the extracellular matrix of the lamina propria. Col-I, Col-III and Col-V staining were light to absent in the smooth muscle layer. Col-IV staining was evenly distributed in the lamina propria and the smooth muscle layer.

In contrast, in animals with PF-induced occlusion in the intramural tube, we identified dense staining of Col-I, Col-III, Col-IV and Col-V in the obstructed area corresponding to the hyalinized zone identified in sections stained with H&E. In these areas, the epithelium was largely absent, and collagen-dense extracellular matrix appeared to replace the epithelium and lamina propria and obscure the tubal lumen.

Col-II was not detected in either control or treated animals.

### 3.3 Collagen staining in the isthmic fallopian tube

**Fig. 2:**
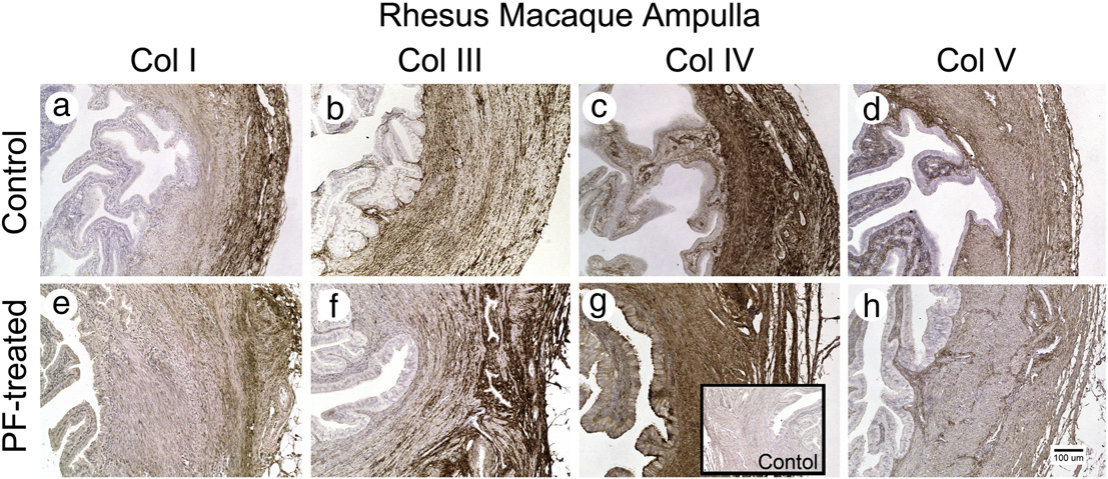
Photographs showing Col-I, Col-II, Col-IV and Col-V staining in rhesus macaque ampulla. Staining for Col-I, Col-III, Col-IV and Col-V was undetectable from the epithelium. No increase in collagen was detected in the PF-treated animals. Control inset shows staining with an irrelevant antibody.

In the ampulla and isthmus, histologic features of normal epithelium were seen with no obstruction in both PF-treated and control animals. Fig. 2 shows collagen staining in the isthmic region of the fallopian tube. Collagen staining showed a similar distribution in PF-treated and untreated controls. There was moderate staining for Col-III, Col-IV and Col-V in the lamina propria. Col-III and Col-V were evenly distributed throughout the muscular layers. Col-I staining was clearly evident in the serosal layers of the tube. Col-II was not detected.

## 4 Discussion

Intrauterine PF administration results in tubal occlusion in most, but not all animals [9], [10]. The reason for these failures remains unclear. The PF-induced tubal occlusion is localized to the intramural region of the tube. The blocked regions contain a “hyalinized amorphous” accumulation of extracellular matrix proteins. Herein, we report that these hyalinized areas stain strongly for Col-I, Col-III, Col-IV and Col-V. Our working hypothesis is that these hyalinized areas represent scar formation resulting from tissue death after sclerosant treatment. Based on historical studies on other agents [13], we propose that exposure to PF results in epithelial and subepithelial injury with the presence of dead cells leading to localized inflammation. The inflammatory phase of the repair process is associated with edema and fibrin formation with cytokine and growth factor release by infiltrating immune cells. If the injury is limited to the epithelium, then reepithelialization can occur restoring the tube to a functional state. However, if the injury involves deeper trauma to the lamina propria, fibroblasts and myofibroblasts are attracted to injured tissue leading to more extensive chronic inflammation with repair involving scar formation and the deposition of extracellular matrix. In skin scar formation, acute granulation tissue contains high levels of Col-III. As the scar matures, this Col-III is slowly degraded and replaced with Col-I [14]. In skin, the high rate of collagen synthesis in wounds lasts up to 6–12 months with degrading scar tissue altering its texture [15]. Turnover of collagen is controlled by various matrix metalloproteinases and tissue inhibitors of matrix metalloproteinases with distinct mechanisms of those regulating inflammation [16]. Therefore, additional studies to examine collagen turnover following PF treatment are warranted as the timing of the process may be critical to the outcome. In the rabbit, tubal obstruction showed a slow process of regeneration that completed over a period of 8 weeks [17]. In our study, Col-III and Col-V staining were stronger than Col-I, indicating that this remodeling process could still be underway.

The pathophysiology immediately following PF-induced tubal damage is not known. This is the first report indicating that PF infusion stimulates collagen deposition. In quinacrine-treated women, the sequence of histopathologic changes was well described [18]. Within days of quinacrine treatment, necrosis of the epithelial lining and acute inflammatory reaction could be observed. Then progressive fibrosis and failure of epithelial regeneration caused tubal occlusion [19].

Our nonhuman primate studies have shown that PF treatment results in epithelial damage and occlusion limited to the intramural fallopian tube [9], [10]. The reason for focal obstruction remains unclear because PF treatment results in PF spillage into the peritoneal cavity [10]. Nonhuman primates provide the most suitable animal model for studies on tubal occlusion because of the presence of the intramural tubal segment that passes through the thick muscular uterine wall of the uterus. This anatomic feature is not present in rodents and domestic species and is uniquely susceptible to damage with sclerosing agents. Intrauterine administration of quinacrine in nonhuman primates resulted in focal scarring in the intramural segment with features identical to those that develop in women treated with quinacrine [18], [20], [21], [22]. Polidocanol is a detergent-based nonionic surfactant, and the cytotoxic effect is dependent on concentration and volume of exposure [23]. Therefore, it is possible that a concentration gradient forms during infusion that lead to the greatest concentration in the intramural tube. It is also possible that the narrow lumen of the intramural tube results in a longer duration of exposure, or increased pressure on epithelial cells as the foam is noncompressible. From a clinical perspective, the absence of nontarget epithelial damage following transcervical administration of PF is reassuring.

It is noteworthy to point out that ectopic pregnancy has been reported for nonhuman primates [24] but is very rare in all animal models compared to women. In women, approximately 10% of ectopic pregnancies occur at locations outside the fallopian tube [11], [25]. Therefore, the high rate of ectopic pregnancy in women is due to characteristics of human embryos and not due to differences in tubal physiology.

In conclusion, PF induced a permanent loss of normal epithelium and accumulation of Col-I, Col-III, Col-IV and Col-V at the site of obstruction. The presence of dense collagen staining indicates the formation of lasting tubal obstructions and strongly supports the further development of PF as a nonsurgical method of permanent female contraception. Additional studies are needed to evaluate the progression of scar formation and factors that may improve the rate of tubal occlusion to near 100%.
